# Occidiofungin inhibition of *Candida* biofilm formation on silicone elastomer surface

**DOI:** 10.1128/spectrum.02460-23

**Published:** 2023-10-10

**Authors:** Rabina Kumpakha, Donna M. Gordon

**Affiliations:** 1 Department of Biological Sciences, Mississippi State University, Mississippi State, Mississippi, USA; Institut Pasteur, Paris, France

**Keywords:** *Candida albicans*, *Candida tropicalis*, biofilms, antifungal agents

## Abstract

Biofilms are the leading cause of clinically acquired fungal infections and contribute to significantly high morbidity and mortality in immunocompromised and hospitalized patients. *Candida* biofilms exhibit increased resistance to currently available antifungal agents that contribute to persistent reoccurring infections and are driving efforts to identify novel fungicidal compounds. The natural product, occidiofungin, is an antifungal compound with demonstrated activity against hyphal morphogenesis in polymorphic *Candida* species. In this study, we use an *in vitro* static biofilm model to demonstrate the efficacy of occidiofungin against *C. albicans* and *C. tropicalis* at all stages of biofilm development, including inhibiting cell dispersal. Consistent with prior findings, we demonstrate that actin organization is altered following occidiofungin exposure to include loss of F-actin cables and accumulation of actin aggregates. Altogether, our results provide strong evidence of the antibiofilm activity of occidiofungin toward *Candida* biofilms and support its potential as a therapeutic for *Candida* infections.

**IMPORTANCE:**

*Candida* are opportunistic fungal pathogens with medical relevance given their association with superficial to life-threatening infections. An important component of *Candida* virulence is the ability to form a biofilm. These structures are highly resistant to antifungal therapies and are often the cause of treatment failure. In this work, we evaluated the efficacy of the antifungal compound, occidiofungin, against *Candida* biofilms developed on a silicone surface. We demonstrate that occidiofungin eliminated cells at all stages of biofilm formation in a dose-dependent manner. Consistent with our understanding of occidiofungin bioactivity, we noted alterations to actin organization and cell morphology following antifungal exposure. Given the challenges associated with the treatment of biofilm-associated infections, occidiofungin exhibits potential as a therapeutic antifungal agent in the future.

## INTRODUCTION

Increasing incidences of *Candida* infections and the emergence of resistance against common antifungal therapies have necessitated the development of novel antifungal compounds with higher efficacy that can target multidrug-resistant strains as well as the polymorphic cells found in biofilms. Many of the clinically approved antifungal compounds target either the fungal cell wall or cell membrane and are highly prone to the development of tolerance and resistance (1). Targeting virulence mechanisms such as yeast-to-hyphae morphological transition and biofilm formation are emerging as potential avenues for the identification of new antifungal compounds with unique mechanisms of action with reduced potential for acquiring resistance (2, 3). Thus, screening of antifungal compounds for their impact on virulence factors is being explored for the development of new and effective antifungals with potential for use in clinical settings for the treatment of *Candida* infections (3 – 5).

The ability to switch from yeast-to-hyphae morphologies is central to the formation of biofilms on biotic and abiotic surfaces. The majority of candidiasis cases are caused by *C. albicans*; however, members of non-*albicans Candida* (NAC) species, especially *C. tropicalis*, are rapidly increasing as leading fungal pathogens in tropical and subtropical environments (6, 7). Compared to non-biofilm (planktonic) cells, cells in a biofilm are more tolerant of exposure to harsh environmental conditions including exhibiting resistance to antimicrobial compounds (8).

Occidiofungin is a natural product produced by the soil bacterium *Burkholderia contaminans* MS14 shown to have broad antifungal activity against a range of fungi including *Candida* species. Structurally, it is a cyclic glycolipopeptide with fungicidal activity that triggers apoptotic cell death (9 – 11). Unlike current clinical antifungal agents that primarily target the integrity of the cell wall or cell membrane, biochemical and cellular evidence supports actin as the biological target of occidiofungin activity (12, 13). In fungi, actin has roles in organelle positioning and inheritance, endocytosis, and polarized cellular growth including hyphal growth in dimorphic *Candida* species (14 – 18). Exposure of actively growing yeast cells to occidiofungin leads to loss of filamentous actin (F-actin) cables (13). Consistent with its impact on actin cables, exposure of *Candida* cells to a sublethal dose of occidiofungin prevents hyphal development in cells induced to undergo morphological transition and if added to cells shortly after switching induction, prevents hyphal elongation (13, 19).

Given our prior findings demonstrating occidiofungin activity against hyphal development, and the known role of actin in hyphal growth and biofilm formation (20 – 22), the efficacy of occidiofungin against biofilm formation in *Candida* species warrants investigation. In this study, we use an *in vitro* static biofilm developed on the surface of medical grade silicone elastomer (SE) as the model for examining occidiofungin efficacy. We find that occidiofungin prevents cell attachment and subsequent biofilm formation for both *Candida albicans* and *Candida tropicalis*. Moreover, occidiofungin effectively eliminates cells present in established *Candida* biofilms and at sublethal concentrations reduces cell dispersal. Confocal analysis of actin organization indicates that occidiofungin alters the actin organization in biofilm cells. Together, our data demonstrate that occidiofungin effectively eliminates cells at all stages of biofilm development and we propose that this is through its disruption of filamentous actin.

## RESULTS

### Occidiofungin inhibited cell attachment during biofilm development

**Fig 1:**
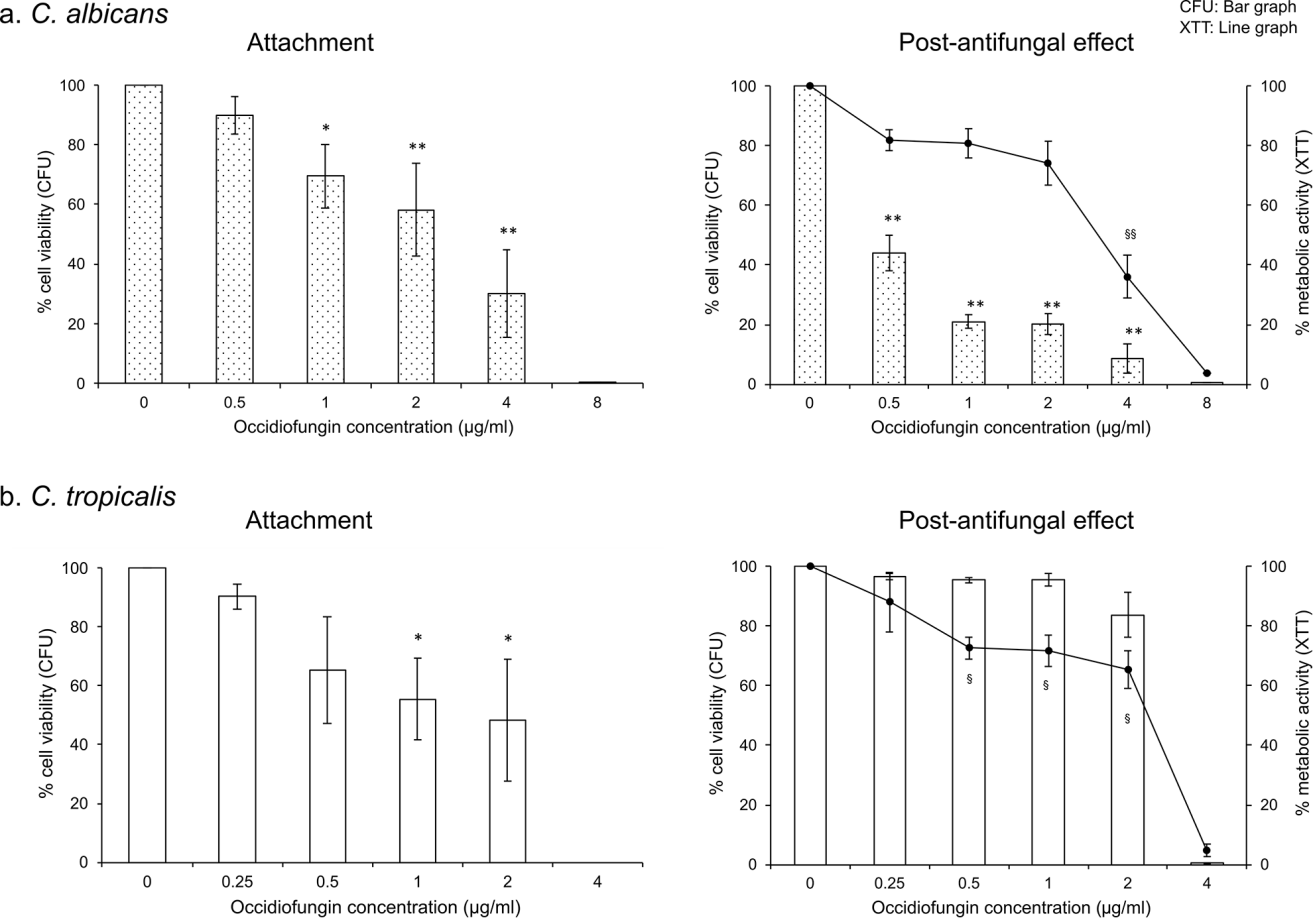
Effect of occidiofungin on *Candida* cell attachment. Relative viable cell number (bar graph) and metabolic activity (line graph) for cells exposed for 90 min to occidiofungin either immediately following treatment (left panel) or 48 h post-treatment (right panel). Data represent percent differences relative to untreated samples for (a) *C. albicans* ATCC 66027 and (b) *C. tropicalis* ATCC 66024. Averages reported for three biological replicates each containing technical triplicates. Error bars represent standard error of the mean. Significant differences, as determined using the post hoc Tukey HSD method, between occidiofungin exposed and untreated cells are indicated; * or ^§^, *P* < 0.05; ** or ^§§^, *P* < 0.01.

To investigate the efficacy of occidiofungin against *Candida* cells present at different stages of biofilm development, we first evaluated its ability to prevent cell attachment to a silicone surface. Cells from *C. albicans* or *C. tropicalis* cultures were incubated with SE discs in the presence of increasing concentrations of occidiofungin for 90 min. Quantification of attached cells immediately following occidiofungin exposure found a dose-dependent reduction in cell number (Fig. 1). The minimum concentration of occidiofungin required to reduce cell number by more than 90% (MBIC_90_) was 8 µg/mL for *C. albicans* and 4 µg/mL for *C. tropicalis* (Fig. 1).

Post-antifungal effects for biofilm cells exposed to occidiofungin during attachment were determined by measuring metabolic activity and viable cell numbers after 48 h of growth. We observed that exposure to occidiofungin during the attachment stage impacted biofilm growth for *C. albicans* but had minimal impact on the final biofilm generated by *C. tropicalis*. For *C. albicans*, a reduction in viable cell number of at least 50% was identified for biofilms formed by cells exposed to even the lowest concentration of occidiofungin tested (Fig. 1a). Unlike *C. albicans*, exposure of *C. tropicalis* cells to occidiofungin during the attachment stage had little impact on subsequent biofilm development as any initial reduction in cell number during attachment was overcome following 48 h of growth (Fig. 1b). Both *C. tropicalis* and *C. albicans* shared a similar general pattern of reduced metabolic activity for biofilms formed from cells exposed to increasingly higher concentrations of occidiofungin. As expected for both strains, exposure to occidiofungin equivalent to its MBIC_90_ during the attachment stage resulted in no biofilm formation by 48 hr.

### Occidiofungin inhibited biofilm formation

**Fig 2:**
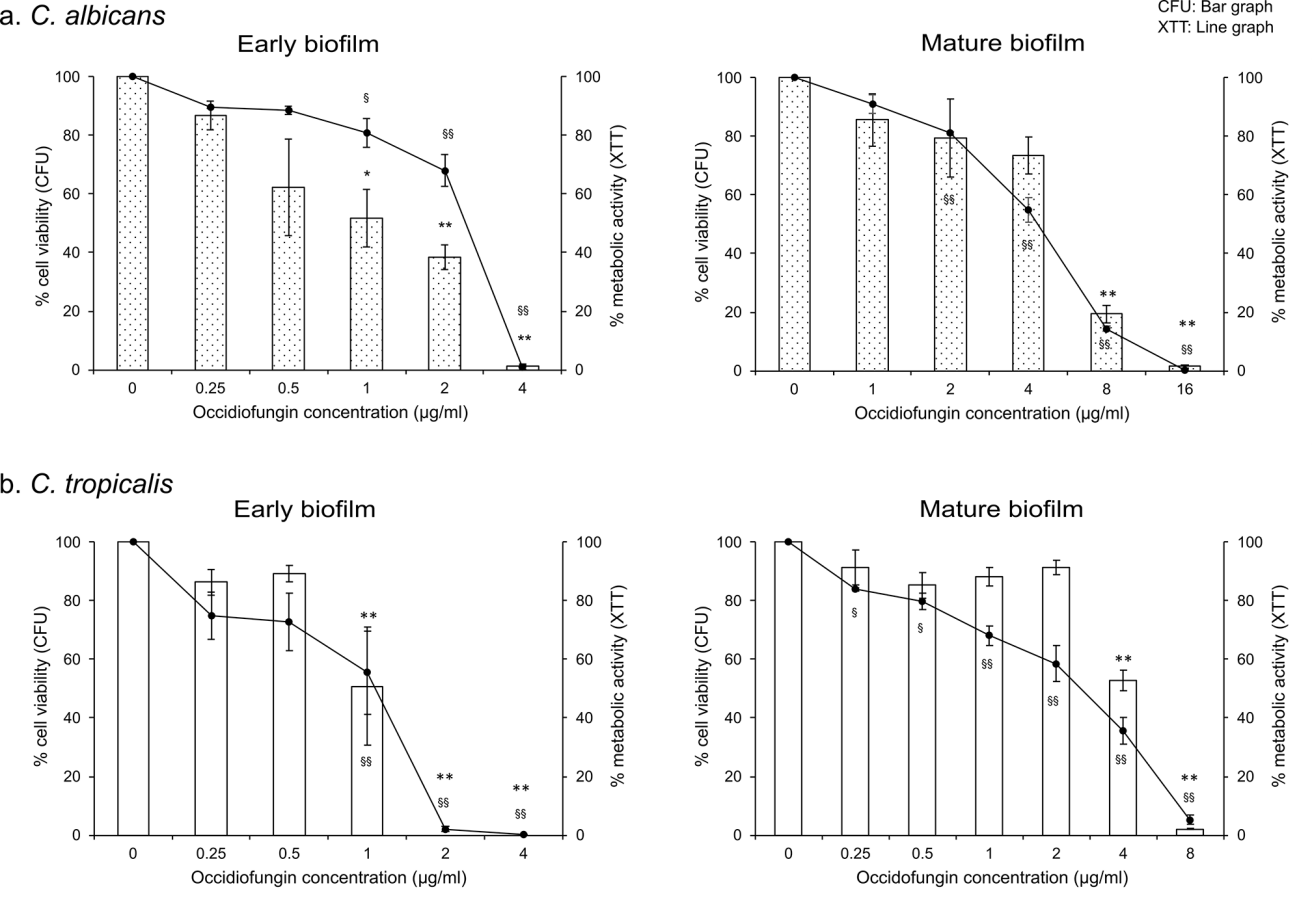
Efficacy of occidiofungin on cells at different stages of biofilm development. The sensitivity of (a) *C. albicans* ATCC 66027 and (b) *C. tropicalis* ATCC 66024 cells to occidiofungin was reported as relatively viable cell number (bar graph) and metabolic activity (line graph). Occidiofungin was added to attached cells and biofilms monitored following a 48 h period (left panel) or added to cells in a 24 h biofilm and monitored following an additional 24 h growth period (right panel). Data from CFU and XTT assays are represented as percent differences relative to untreated biofilm cells with the average and standard error for three biological replicates each containing technical triplicates. Significant differences, as determined using the post hoc Tukey HSD method, between untreated and occidiofungin exposed biofilms are indicated; * or ^§^, *P* < 0.05; ** or ^§§^, *P* < 0.01.

To determine whether occidiofungin was effective at later stages of biofilm development, the antifungal was added to cells following attachment, and biofilm was allowed to develop for 48 h. Like that found during the attachment stage, a trend of dose-dependent reduction in relative metabolic activity and viable cell number was observed for biofilms of *C. albicans* (Fig. 2a). The minimum occidiofungin concentration required for complete inhibition of biofilm growth was 4 µg/mL, twofold lower than that found for eliminating cells at the attachment stage. Similarly, a twofold lower dose of occidiofungin was required to prevent biofilm formation by *C. tropicalis* (MBIC_90_; 2 µg/mL) (Fig. 2b and Table 1). For both *Candida* species, 0.5× MBIC_90_ concentrations of occidiofungin reduced both metabolic activity and cell number by 50%. Lower concentrations had only a minimal impact on *C. albicans* and no significant impact on the final biofilm formed by *C. tropicalis*.

**TABLE 1:** Minimum occidiofungin required to eliminate cells in *Candida* biofilm^*b*^

Strain	Biofilm stage	Cell number (CFU/mL)^*a*^	MBIC_90_ (µg/mL)
*C. albicans*	Attachment Early biofilm Preformed biofilm	1 ± 0 × 10^6^ 1.1 ± 0.1 × 10^5^ 2.2 ± 0.5 × 10^6^	8 4 16
*C. tropicalis*	Attachment Early biofilm Preformed biofilm	1 ± 0 × 10^6^ 1.0 ± 0.3 × 10^4^ 9.4 ± 0.9 × 10^6^	4 2 8

^*a*^ Starting cell number at the time of occidiofungin addition for each developmental stage.

^*b*^ Data presented from three independent experiments.

Next, we measured the impact of occidiofungin on preformed biofilms. Established biofilms at 24 h of development were treated with a range of occidiofungin (0–32 μg/mL) and the impact was measured following a 24 h growth period. For both *C. albicans* and *C. tropicalis*, a dose-dependent reduction in metabolic activity and viable cell number was found for occidiofungin-exposed biofilms. The minimum concentration of occidiofungin required to eliminate cells in an established biofilm was higher than that required for cells during attachment and early biofilm stages of formation (Fig. 2; Table 1). For *C. albicans*, a 90% reduction in biofilm cell number was achieved with 16 µg/mL occidiofungin followed by an 80% reduction with 8 µg/mL (Fig. 2a). Metabolic activity measurements found a similar sensitivity profile.

By contrast, the *C. tropicalis* biofilm showed higher sensitivity toward occidiofungin with 8 µg/mL occidiofungin eliminating cells in the biofilm and 4 µg/mL reducing cell number by more than 50% (Fig. 2b). Lower concentrations resulted in less than 10%–20% reduction in viable cell number.

### Short-term exposure reduced biofilm cell viability

**Fig 3:**
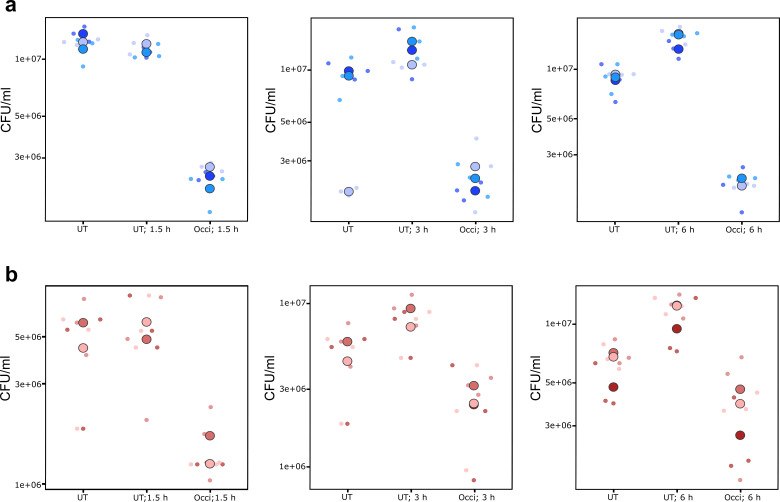
Assessment of short-term exposure of preformed biofilm to occidiofungin. Exposure of *C. albicans* ATCC 66027 and *C. tropicalis* 66024 preformed biofilms to 0.5× MBIC_90_ occidiofungin. Viable cell numbers following 1.5 h, 3 h, and 6 h treatment of antifungal were analyzed and presented as CFU/mL for (a) *C. albicans* and (b) *C. tropicalis*. Data are presented from three independent experiments each containing three biofilm disks. The data from each biofilm (small dots) and average from three replicates (large dots) are shown. (c) Relative percent reduction in metabolic activity and viable cell number following short-term exposure to 0.5× MBIC_90_ occidiofungin for different time periods.

As prior work demonstrated that cellular response to occidiofungin could be detected within 30 min of exposure (9, 19), we next examined the short-term impact on cells of a preformed biofilm 1.5 h, 3 h, and 6 h after occidiofungin addition. For both *C. albicans* and *C. tropicalis*, a 0.5× MBEC_90_ dose of occidiofungin significantly reduced the cell number in a 24-h biofilm, regardless of when assayed. However, this reduction may underestimate occidiofungin efficacy given the increase in cell number detected with the transfer of biofilms into fresh media at the time of occidiofungin addition (Fig. 3a and b). Compared to an untreated control biofilm, occidiofungin treatment resulted in an average 70%–88% reduction in viable cell number after 1.5 h, 3 h, or 6 h of addition for *C. albicans* and a 70%–79% reduction for *C. tropicalis* (Table 2). In addition, the metabolic activity of biofilm cells was reduced following occidiofungin exposure by 78%–87% for *C. albicans* and 70%–77% for *C. tropicalis* (Table 2).

**TABLE 2:** Biofilm reduction with short-term exposure to 0.5× MBIC_90_ occidiofungin

	Occidiofungin exposure time (h)	Relative percent reduction (%)	
		Metabolic activity	Cell viability
*C. albicans*	1.5 3 6	70.54 ± 4.92 76.23 ± 3.17 87.58 ± 2.36	78.83 ± 1.87 80.54 ± 5.81^*a*^ 87.20 ± 1.9
*C. tropicalis*	1.5 3 6	71.09 ± 2.37 79.66 ± 6.44 70.51 ± 4.95	77.36 ± 1.32 69.88 ± 6.52^*a*^ 77.78 ± 3.85

^*a*^ Statistically significant difference between corresponding data for *C. albicans* and *C. tropicalis*, *P* < 0.05.

### Occidiofungin altered the morphology of biofilm cells

**Fig 4:**
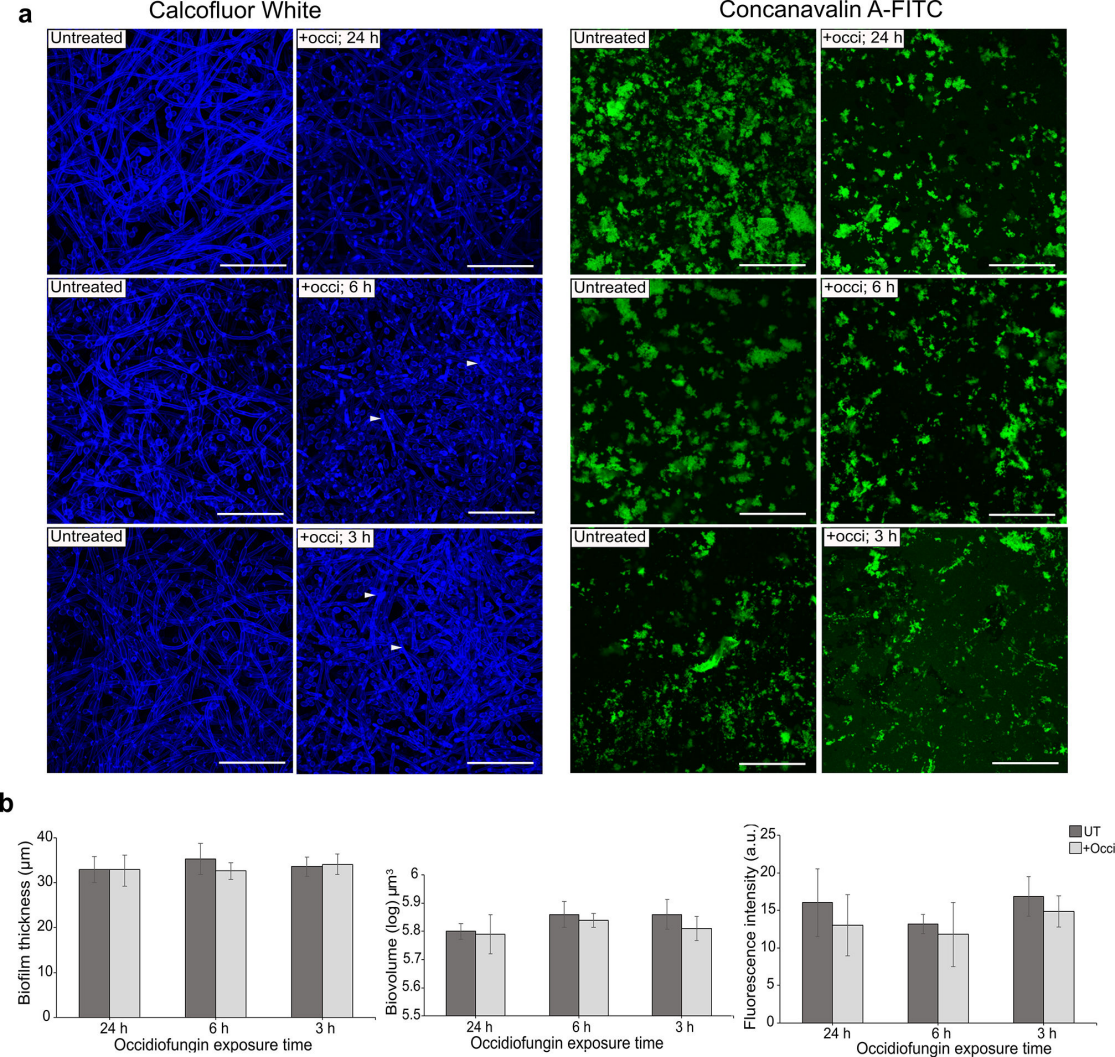
Occidiofungin exposure alters the morphology of biofilm cells. Organization of *C. albicans* ATCC 66027 cells in a preformed biofilm following growth in RPMI media (untreated) or RPMI with 0.5× MBIC_90_ occidiofungin (+Occ) for 24 h, 6 h, or 3 h. (a) Representative images of biofilms co-stained with Calcofluor White (left panel) and Concanavalin A-FITC (right panel) to visualize cell morphology and extracellular matrix. Images are displayed as maximum intensity projections of 3D z-stacks. White arrowheads indicate areas of enhanced chitin staining. Size bar: 50 micron. (b) Biofilm thickness, biovolume, and mean intensity fluorescence for ECM (Concanavalin A-FITC) data are reported for untreated (dark bars) and occidiofungin treated (light bars) biofilms with data collected from 10 different fields obtained for each of 3 independent biological replicates per condition. Data represent the average and standard error. No significant differences between untreated and occidiofungin-treated biofilms were found using the post hoc Tukey HSD method.

Changes in biofilm structure resulting from short-term exposure to occidiofungin were determined using a confocal laser scanning microscope (CLSM). Biofilm treated with 0.5× MBIC_90_ occidiofungin for 1.5 h, 3 h, and 6 h were analyzed for morphological changes following staining with Calcofluor White (CW) and Concanavalin A FITC (Con A-FITC) to visualize chitin and extracellular matrix material, respectively. Whereas untreated 24-h *C*. *albicans* biofilm consisted primarily of true hyphae embedded within extracellular matrix material (Fig. 4), untreated *C. tropicalis* biofilm contained mainly cells in the yeast form (Fig. S1). Treatment of *C. albicans* biofilm with 0.5× MBIC_90_ dose of occidiofungin for 1.5 h, 3 h, or 6 h resulted in fewer hyphal cells and more pseudohyphae, abnormal hyphae, or yeast-form cells compared to untreated control. *C. tropicalis* biofilm exposed to occidiofungin, on the other hand, did not exhibit distinct alterations in cell morphology (Fig. S1a). However, in *C. albicans*, cells in treated biofilms were observed to have increased deposition of chitin along the hyphal length relative to untreated control biofilms (Fig. 4a; Fig. S2). Analysis of biofilm cells 24 h after occidiofungin addition found minimal evidence of cells with altered morphology (Fig. 4a; Fig. S2). Quantification of other biofilm parameters for *C. albicans* revealed that occidiofungin had no impact on extracellular matrix (ECM), overall biofilm thickness, or biovolume (Fig. 4b; Table 3). By contrast, *C. tropicalis* biofilm exhibited reduced ECM with no change in biofilm thickness or biovolume following occidiofungin treatment (Fig. S1b).

**TABLE 3:** Biofilm parameters for untreated and occidiofungin-exposed biofilms

Strain	Treatment	Exposure time (h)	Thickness (µm)	Biovolume (log µm^3^)	Dead cells/biovolume (%)
*C. albicans*	Untreated	3	35.1 ± 5.6	5.7 ± 0.16	8.0 ± 4.5
		24	37.1 ± 3.1	5.5 ± 0.1	12.0 ± 1.5
	Occi treated	3	37.6 ± 3.8	5.8 ± 0.1	80.0 ± 4.2^*a*^
		24	36.2 ± 3.2	5.6 ± 0.1	79.0 ± 6.2
*C. tropicalis*	Untreated	3	18.0 ± 1.3	5.5 ± 0.13	12.0 ± 1.5
		24	18.8 ± 0.5	5.5 ± 0.13	7.6 ± 2.6
	Occi treated	3	16.9 ± 0.3	5.6 ± 0.1	63.0 ± 5.1^*a*^
		24	18.6 ± 1.3	5.4 ± 0.05	74.0 ± 5.6

^*a*^ Significant difference as determined using the post hoc Tukey HSD method, between corresponding data for *C. albicans* and *C. tropicalis*; *P* < 0.01.

### Occidiofungin reduced the release of viable cells from a biofilm

**Fig 5:**
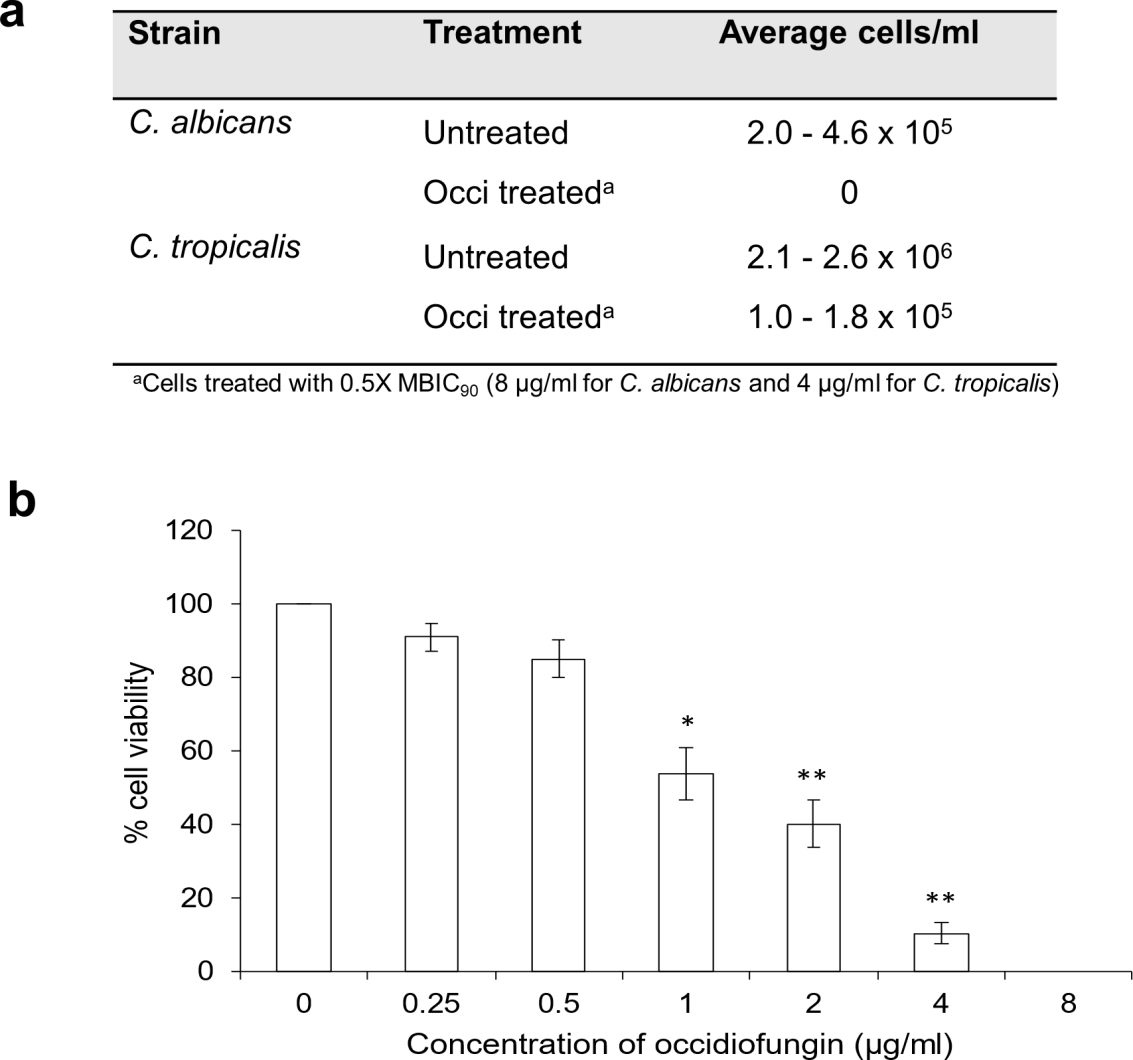
Occidiofungin reduces cells released from a biofilm. (a) Average CFU/mL of dispersed cells from untreated and 0.5× MBIC_90_ occidiofungin-treated *C. albicans* ATCC 66027 and *C. tropicalis* ATCC 66024 biofilms. (b) Dispersed cells from *C. tropicalis* biofilm treated with increasing concentration of occidiofungin presented as % viable cells. Data presented as average and standard error from three independent experiments. Significant differences, relative to untreated biofilm, were determined using the post hoc Tukey HSD method. *P < 0.05; **P < 0.01.

To determine whether exposure of biofilm cells to occidiofungin impacted the number of cells released from a biofilm, cells dispersed from a biofilm following 24 h of growth were quantified. We found that the number of viable cells released from control biofilms differed between *C. tropicalis* and *C. albicans* with approximately 10-fold higher cell number for *C. tropicalis* compared to *C. albicans* biofilm (Fig. 5a). Treatment with 0.5× MBIC_90_ occidiofungin for 24 h resulted in no viable cells found in spent media from *C. albicans* biofilm while significantly reducing (~10-fold) the number from *C. tropicalis*. As previously reported (23, 24), microscopic analysis confirmed the morphology of released cells as primarily in yeast form with few pseudohyphal cells (Table S1).

As cells dispersed from a biofilm can seed growth at a new location, we next measured the efficacy of occidiofungin against biofilm-released cells. Susceptibility assays for *C. albicans-* and *C. tropicalis*-dispersed biofilm cells found that the minimum inhibitory concentration was 2 µg/mL for both *Candida* strains, similar to previous findings for planktonic yeast-form cells (19). We were unable to determine the efficacy of occidiofungin against previously exposed biofilm cells as the treatment regime reduced the number of released cells below that required for susceptibility assays.

To further evaluate the impact of occidiofungin on cell dispersal from biofilms, a range of occidiofungin concentrations were tested, with viable cell release quantified by CFU. A concentration-dependent reduction in released cell number was detected for biofilms exposed to occidiofungin concentrations as low as eightfold below that of the biofilm MBEC_90_ (Fig. 5b). Interestingly, concentrations that had no apparent impact on cell viability within the biofilm were still able to significantly reduce the number of cells released from a biofilm (compare Fig. 2b with Fig. 5b). However, there is a threshold effect as lower concentrations of occidiofungin (≤0.5 µg/mL) led to no change in dispersed cell numbers.

### Occidiofungin-killed cells were retained within a biofilm

**Fig 6:**
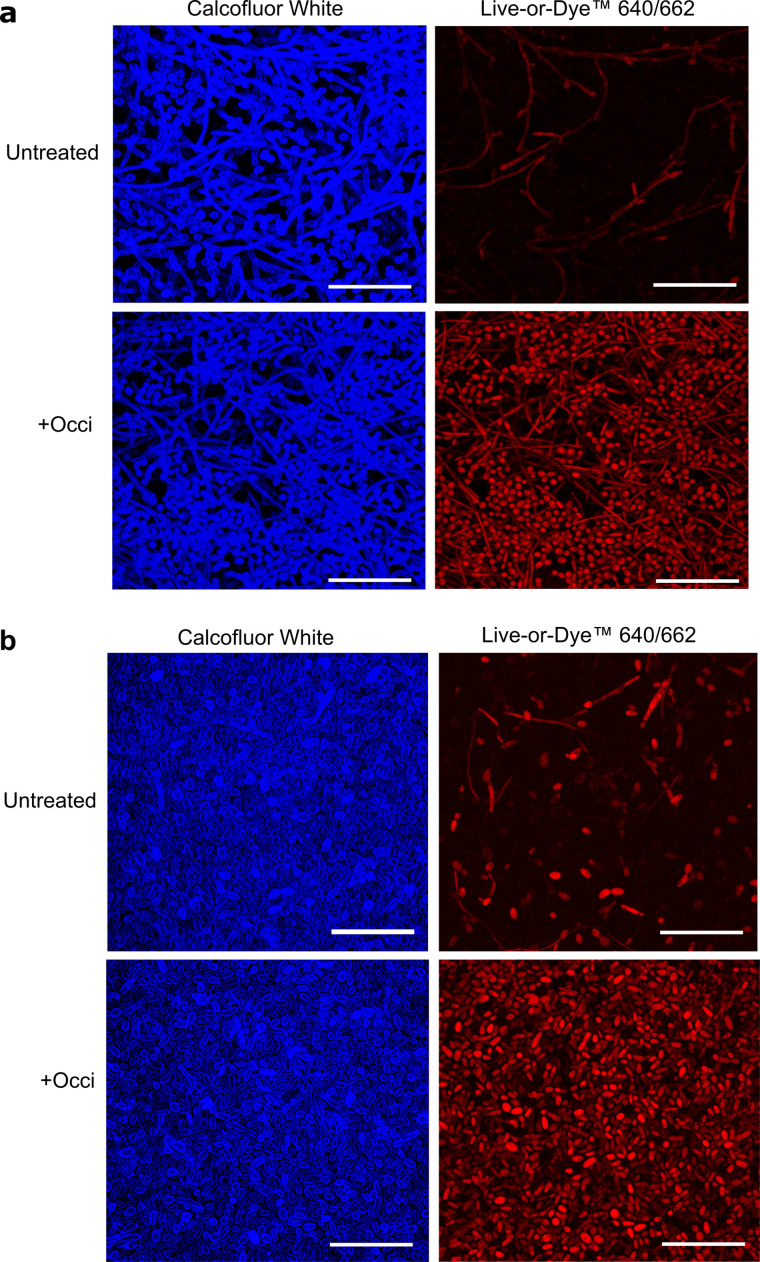
Occidiofungin killed cells are retained within the biofilm. Representative CLSM images of untreated and 3 h occidiofungin treated for (a) *C. albicans* ATCC 66027 and (b) *C. tropicalis* ATCC 66024 biofilm stained with Calcofluor White (left panel) and Live-or-Dye 640/662^TM^ viability dye (right panel) to visualize total and dead cells, respectively. Size bar: 50 microns.

The finding of little change in biofilm volume and thickness following occidiofungin exposure despite the >70% reduction in viable cell number suggested that occidiofungin-killed cells were retained within the biofilm structure. To test this, we determined the percentage of dead cells by biovolume in untreated and occidiofungin-treated biofilms relative to total cell biovolume using Live-or-Dye viability stain and Calcofluor White stain, respectively (Fig. 6). *C. albicans-* and *C. tropicalis*-untreated biofilms were found to contain 8% or 12% of their volume as dead cells compared to occidiofungin-treated biofilms where 75% or 63% of the biovolume was occupied by dead cells after only 3 h exposure. In both cases, there was no additional increase in the proportion of the biofilm occupied by dead cells 24 h following occidiofungin addition (Table 3).

### Occidiofungin altered actin organization in switching and biofilm cells

**Fig 7:**
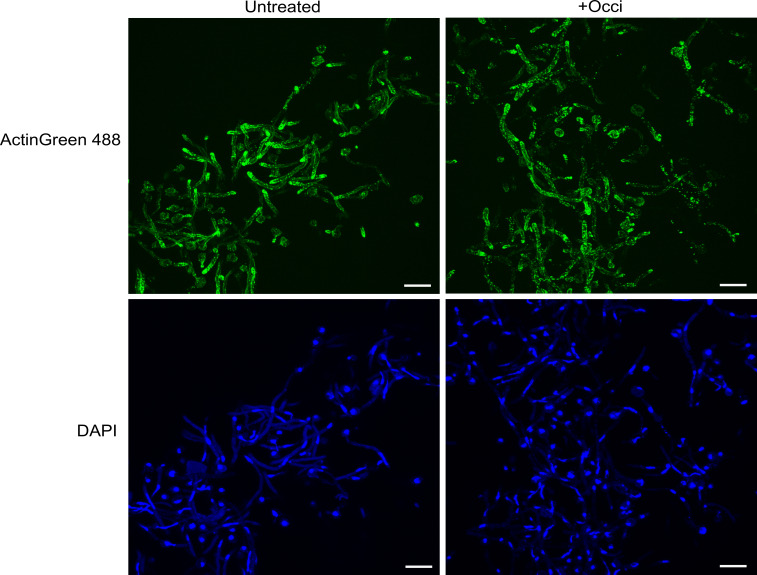
Loss of actin cables in morphologically switching cells exposed to occidiofungin. *Candida albicans* ATCC 66027 cells induced to undergo hyphal morphogenesis were left untreated (left) or exposed to 0.5× MIC (0.25 µg/mL) occidiofungin (right) for 2 h. Representative CLSM images of cells stained with DAPI (bottom panel) to visualize nuclei and ActinGreen 488 stain (top panel) to visualize actin are shown. Size bar: 10 microns.

Prior work with occidiofungin demonstrated that the antifungal induces loss of actin cables in yeast-form cells and blocks hyphal formation and extension in morphologically switching *C. albicans* cells (13, 19). Here, we extend these findings to identify the effect of occidiofungin on actin organization in cells undergoing hyphal morphogenesis and cells within an intact biofilm using CLSM. For untreated hyphal cells induced in Spider medium for 2 h, actin was found to extend as long filamentous cables along the length of hyphae and accumulate in actin patches at hyphal tips (Fig. 7). With short-term occidiofungin exposure, loss of actin cables was observed in the majority of cells with a concomitant accumulation of F-actin aggregates throughout the hyphal length (Fig. 7). These aggregate structures are consistent with prior observations identified for actin organization in occidiofungin-exposed yeast-form cells (13).

**Fig 8:**
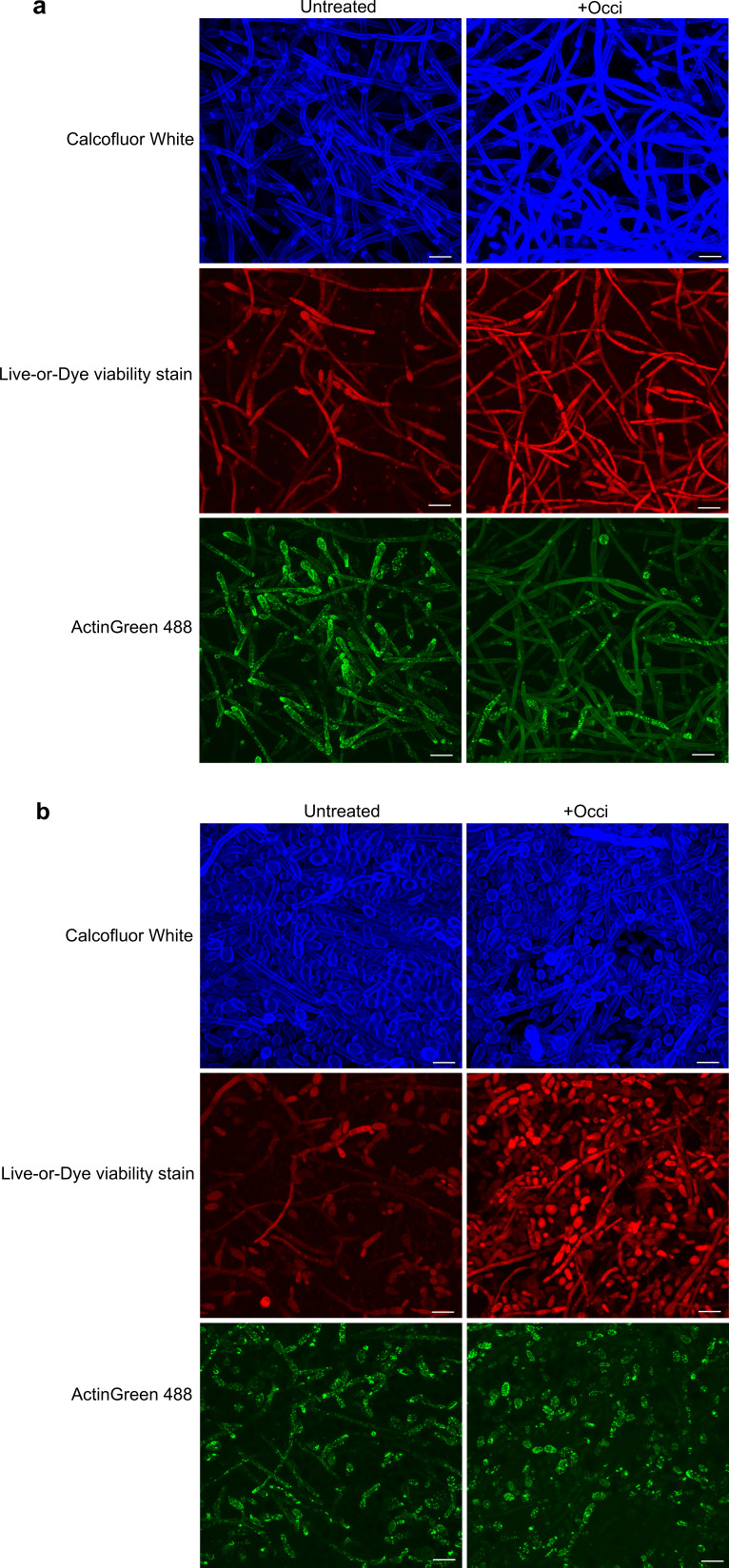
Loss of actin cables in occidiofungin exposed biofilm cells. Preformed 24-h biofilm of *Candida* ATCC 66024 cells grown in RPMI media were left untreated (left) or treated with 0.5× MBIC_90_ occidiofungin (right) for 3 h. (a) *C. albicans* ATCC 66027 (0.5× MBIC_90_; 8 µg/mL) and (b) *C. tropicalis* ATCC 66024 (0.5× MBIC_90_; 4 µg/mL) biofilm cells stained with Calcofluor White (top panel), Live-or-Dye viability stain (middle panel), and ActinGreen 488 (bottom panel) to visualize cell wall chitin, dead cells, and actin organization, respectively. Representative CLSM images of biofilm cells are shown. Size bar: 10 microns.

Actin organization in untreated *C. albicans* biofilm cells differed from that found in switching cells with cells in a biofilm found to have fewer F-actin cables. Instead, actin was found distributed as a large punctate structure that concentrated at the growing tip (Fig. 8a). Occidiofungin exposure resulted in the loss of actin cables and the accumulation of larger actin aggregates. Notably, many of the hyphal cells lacked organized actin structures altogether and instead had diffuse actin staining along the hyphal length (Fig. 8a). Similar accumulation of actin aggregates was identified for *C. tropicalis* biofilm cells (Fig. 8b).

To determine whether the loss of actin structures identified in cells in an occidiofungin-treated biofilm was a result of large-scale protein degradation, the steady state level of actin was measured by immunoblot analysis. Relative to a control protein, we found no loss in actin protein levels in biofilm cells following short-term exposure to occidiofungin (Fig. S3).

## DISCUSSION

The ability of *Candida* to switch from yeast to hyphal form and develop as a biofilm is considered important for its pathogenicity. Although echinocandins, azoles, and amphotericin are considered the gold standard for the treatment of *Candida* infections, biofilm-related infections exhibit resilience to these antifungals (25 – 27). The limited arsenal of antifungals that target biofilm-associated infections is therefore driving the search for alternative antifungal compounds with efficacy against fungal cells found within these structures (28 – 30).

The present study focused on a novel antifungal compound, occidiofungin, produced by the soil bacteria *Burkholderia contaminans* MS14 with inhibitory activity against the *Candida* yeast-to-hyphal transformation likely through its impact on filamentous actin (13, 19). Since the pathogenicity and ability of *Candida* to form biofilm is attributed to their morphological transition, we focused our investigation on the efficacy of occidiofungin in the prevention and elimination of *Candida* biofilms using an *in vitro* biofilm model. For comparison, we included *C. albicans* and *C. tropicalis*, both shown to develop biofilms with different characteristics with regard to cell morphology and biofilm organization (31 – 34). Our studies found that occidiofungin is effective at eliminating cells at all stages of biofilm development for both *C. albicans* and *C. tropicalis*. As expected, the amount of occidiofungin required to eliminate cells was correlated with the number of cells present at each stage of biofilm formation with the highest concentration (16 µg/mL) needed for preformed biofilms. We also found that biofilms formed by *C. tropicalis* were more sensitive to occidiofungin compared to *C. albicans*, requiring twofold lower occidiofungin at every stage of formation. This difference in sensitivity is consistent with our prior work examining occidiofungin susceptibility for yeast and switching form cells for these *Candida* strains (19).

Subtle stage-specific differences in susceptibility to sublethal concentrations of occidiofungin were noted for the two *Candida* species. Of note was the occidiofungin post-antifungal effect identified for *C. albicans* biofilm cells that were essentially absent for *C. tropicalis*. This subtle difference in response to occidiofungin may be due to differences in the developmental program of biofilm formation between the species or the finding that cells undergoing hyphal morphogenesis in *C. albicans* are more sensitive to occidiofungin than their yeast form (19).

Similar to published reports, we found *C. albicans* mature biofilm consisted primarily of hyphal cells with some pseudohyphae and yeast cells (35) while the biofilm formed by *C. tropicalis* had a large number of densely packed yeast cells (36, 37). Both biofilms had a similar percentage of their biovolume occupied by dead cells and ECM materials. Prior studies on static biofilm formed by *C. tropicalis* found them to be more resistant to antifungals such as amphotericin B and fluconazole due to the inhibitory effect extracellular matrix components have on antifungal penetration of mature biofilm (31, 38). No such enhanced resistance to occidiofungin was identified for *C. tropicalis* relative to *C. albicans* at any stage of biofilm development. However, the twofold higher dose of occidiofungin required to eliminate an approximately equivalent number of cells in a mature stage biofilm relative to that during cell attachment suggests that differences in cell morphology, cell density, or the presence of extracellular material negatively impact the antifungal activity of occidiofungin.

At a structural level, changes in cellular morphology were evident with short-term exposure to occidiofungin that included an increase in chitin staining. This is consistent with prior work on yeast-form cells showing that exposure to occidiofungin increased cell wall chitin levels through activation of the cell wall integrity pathway (9). Alterations to cell morphology were less obvious when biofilms were analyzed 24 h following the addition of occidiofungin. Outside these observations, no significant changes were found in biofilm biovolume and thickness following occidiofungin exposure despite the reduction in viable cell numbers. This similarity in biofilm parameters was found to be due to the retention of dead cells within the biofilm structure. Under the conditions used for biofilm formation, untreated biofilms were found to contain 8%–12% dead cells, which is in line with findings from one study (39), but higher than that reported by other authors (40). This discrepancy may reflect differences in the growth conditions used for biofilm formation or the approach used for data analysis and measurement. Nevertheless, in the present study, no significant differences were found in the percentage of dead cells in *C. albican*s and *C. tropicalis* biofilms regardless of whether they were analyzed 3 h or 24 h following occidiofungin addition. However, *C. tropicalis* biofilms were found to have significantly fewer dead cells relative to biofilm biomass 3 h after occidiofungin addition compared to that of *C. albicans*. This lower percentage of dead cells may be due to the presence of densely packed yeast cells within a thinner biofilm coupled with a short time of antifungal exposure which may reflect penetration differences between *Candida* species for the antifungal.

Cells dispersed from a biofilm are reported to be morphologically in yeast form but developmentally distinct from biofilm cells and planktonic yeast cells (23). Here we demonstrate that occidiofungin treatment of biofilms eliminated the accumulation of dispersed cells in the spent media of *C. albicans* biofilm and reduced the dispersed cell numbers by 73% for *C. tropicalis*. The ability of occidiofungin to eliminate most but not all of the cells released from *C. tropicalis* biofilm may reflect differences in the proportion of cells being dispersed in the environment over time or the cellular organization of biofilms compared to *C. albicans*. Our finding of approximately 10-fold higher cell number in spent media of *C. tropicalis* biofilm compared to *C. albicans* biofilm despite a similar number of cells present in the biofilm of each would support this interpretation. In addition, the presence of primarily yeast-form cells in *C. tropicalis* biofilm would be expected to provide a larger source of yeast-form cells for release into the media compared to the hyphal-rich *C. albicans* biofilm (24, 33, 41, 42). Furthermore, the dispersal of cells was eliminated at concentrations of occidiofungin that showed no impact on biofilm cells. These findings may reflect susceptibility differences between dispersed and biofilm cells or suggest a role for occidiofungin in inhibiting the developmental program that gives rise to dispersed cells.

Actin is important for polarized growth of hyphal cells and biofilm development as studies have shown that actin cytoskeleton dynamics are responsible for activation of signaling pathways required for filamentation and actin regulatory proteins have been identified for their role in biofilm formation (20, 43 – 45). Here, we have extended our prior findings demonstrating that occidiofungin inhibits both hyphae formation and hyphae extension to confirm loss of actin organization in hyphal cells following short-term exposure (13, 19). These changes in actin organization were also found to occur in cells within a biofilm. To our knowledge, this is the first study reporting on the actin organization in *Candida* biofilm cells. The dependency on actin for polarized growth and biofilm formation may differ from species to species which may contribute to differences in occidiofungin activity between *Candida* species. This may explain the differential activity of occidiofungin against *C. albicans* and *C. tropicalis* biofilm cells (19). Occidiofungin also inhibits and eliminates biofilm growth at all developmental stages. We propose that this inhibition or elimination of biofilm is through its disruption of actin organization which ultimately triggers apoptosis (9, 13).

Finally, occidiofungin’s interference with the ability of *Candida* species to establish a biofilm at all stages of development supports its promise as a potential candidate for the elimination of *Candida* biofilm-related infections. However, as this study was done using an *in vitro* biofilm model, occidiofungin efficacy in an *in vivo* model of biofilm formation will be the next step to confirming the antifungal potential of occidiofungin in a clinical setting.

## MATERIALS AND METHODS

### Yeast strains and growth conditions

*C. albicans* ATCC 66027 and *C. tropicalis* ATCC 66024 cells were revived from −80°C glycerol stocks with growth on yeast peptone dextrose plates (YPD; 2% peptone, 1% yeast extract, 2% glucose, 2% agar) at 30°C for 48 h. Revived fungal cells were used directly for inoculation into liquid YPD in preparation of biofilm formation. Biofilm formation was carried out in RPMI-1640 media (with 0.3 g/L L-glutamine, 0.2% glucose, 165 mM MOPS, pH 6.93) at 37°C (Gibco)

### Biofilm formation on SE disks

Medical grade silicone elastomer sheets were cleaned with soap and hot water using a soft bristle toothbrush and rinsed twice with hot water as instructed by the manufacturer (Bioplexus). Cleaned sheets were placed between lint-free paper and cut into 11 mm diameter disks using a #5 cork borer (Sigma). Disks, in a glass bottom dish, were sterilized for 30 min at 121°C before use.

Biofilms were established on SE disks in a flat bottom tissue culture treated 24-well plate as previously described (46). Briefly, prior to use, sterile SE disks were incubated in RPMI media overnight at 37°C. Cells from cultures grown 24 h in YPD were isolated by centrifugation at 4°C and 5,000× *g*, washed twice with 1× phosphate-buffered saline (PBS), and resuspended in prewarmed RPMI media to obtain a final cell density of 1 × 10^6^ cells/mL. The diluted cell suspension (800 µL) was added to pre-incubated disks and cells allowed to attach for 90 min at 37°C with gentle shaking at 75 rpm. Media and unattached cells were removed with two washes in 1× PBS, fresh pre-warmed RPMI media was added, and the plate returned to 37°C with 75 rpm shaking to allow biofilm formation for 24 h or 48 h.

### Antifungal preparation

Occidiofungin was a gift from Dr. James Leif Smith, Department of Biological Sciences, Texas A&M University and was isolated from *Burkholderia contaminans* strain MS14 as described previously (10). Stock solutions of 10 mg/mL occidiofungin were prepared in 100% dimethyl sulfoxide and stored at −20°C until use. Antifungal dilutions were generated by twofold serial dilution in pre-warmed RPMI media and then added to biofilm cells in 24-well plates.

### Biofilm treatment at different stages of development

The minimum concentration of occidiofungin required to inhibit (MBIC_90_) or eradicate (MBEC_90_) biofilm cells was determined for early biofilm and mature biofilm, respectively. For cell treatment during the attachment stage, occidiofungin (8–0.25 µg/mL) was added to cells (10^6^ cells/mL) in RPMI media and transferred to wells containing SE disks for the 90 min attachment period. Biofilm cells were analyzed immediately following the 90 min attachment period or, for post-antifungal studies, the biofilm was washed twice in 1× PBS and placed in a new microtiter well with fresh RPMI media for 48 h. For experiments using early biofilm, occidiofungin (16–0.25 µg/mL) in RPMI media was added to cells immediately following attachment to SE disks, and the biofilm was allowed to develop for 48 h in the presence of the antifungal. For experiments using preformed biofilm, occidiofungin (32–1 µg/mL) in RPMI media was added to cells in a 24-h biofilm followed by a 24 h period of growth. The lowest concentration of antifungal that eliminated 90% biofilm formation was determined using XTT assay (MBIC_90_ or MBEC_90_); disks with no cells were used as a negative control. For all experiments, control biofilms were treated with an equivalent volume of DMSO (vehicle control). Each plate included triplicate testing of antifungal concentrations and each experiment included three independent biological replicates.

### XTT assay

To measure metabolic activity, biofilms were washed twice with 1× PBS and transferred to a new 24-well plate. A 175 µL volume of XTT reagent mixture (Biotium) was added to wells of 24-well plates containing biofilms in 700 µL 1× PBS. Plates were incubated at 37°C in the dark for 45 min with gentle shaking at 75 rpm. The reaction solution from each well (80 µL) was transferred to a 96-well plate in triplicate, and absorbance was measured at 490 nm using a microplate reader (Bio-Rad Laboratories). The biofilm activity is reported as percent metabolic activity relative to control using the equation:


$$\%\;metabolic\;activity\;in\;biofilm=1-[\frac{OD_{control;490nm}-OD_{treated;490nm}}{OD_{control;490nm}}]\times 100$$

### CFU assay

Following the XTT assay, disks were washed with 1× PBS and transferred to a 5 mL microfuge tube containing 1 mL of 1× PBS. Disks were subjected to 3 rounds of 1 min bath sonication followed by 30 s vortexing to disrupt the biofilm structure. The resulting cell suspension was fivefold serially diluted in 1× PBS and 50 µL from select dilutions spread on YPD plates in triplicate. After 48-h growth at 30°C, colonies were counted and plates with 30–300 colonies were used for analysis. Data are reported as either the log_10_ CFU/mL for all samples or as the relative percent cell viability as determined using the following equation:


$$Relative\;percent\;cell\;viability=1-[\frac{CFU_{untreated}-CFU_{treated}}{CFU_{untreated}}]\times 100$$

### Quantification and susceptibility testing of biofilm-dispersed cells

Biofilms were developed on SE disks for 24 h or 48 h in the presence and absence of 0.5× MBIC_90_ of occidiofungin as described above. The spent media containing dispersed cells were collected from untreated and treated biofilms, washed with 1× PBS, and quantified by CFU assay. For antifungal susceptibility of dispersed cells, spent media from 48 h *C*. *albicans* and *C. tropicalis*-untreated biofilms were collected and diluted to 1 × 10^4^ cells/mL in RPMI media. Susceptibility assays in 96-well microtiter format were performed as described previously (19).

### Calcofluor white and concanavalin A-FITC

Processing of biofilm cells for confocal microscopy was carried out as described previously (35, 47). Briefly, biofilms grown on SE disks for 24 h were placed in fresh RPMI media or treated with 0.5× MBIC_90_ dose of occidiofungin in RPMI for 1.5 h, 3 h, 6 h, and 24 h. Following treatment, biofilms were transferred into 4% formaldehyde solution for 30 min, washed with 1× PBS, and stained with 50 µg/mL CW (Sigma) and 25 µg/mL Con A-FITC conjugate solution overnight at 37°C in the dark.

### Live-or-Dye viability

Preformed biofilms were exposed to 0.5× MBIC_90_ of occidiofungin for 3 h or 24 h. Untreated and occidiofungin-treated biofilms were stained with Live-or-Dye™ 640/662 Fixable Viability Stain (Biotium) for 30 min in 1× PBS. Stained biofilms were washed and fixed in a 4% formaldehyde solution for 30 min. Biofilms were washed in 1× PBS and counterstained with CW as described above.

### Actin

Actin was visualized in morphologically switching *C. albicans* ATCC 66027 cells and biofilm cells by confocal microscopy using ActinGreen 488 ReadyProbes Reagent (ThermoFisher). Briefly, cells induced to undergo morphological switching in Spider media at 37°C for 2 h were exposed to 0.5× MIC occidiofungin (0.25 µg/mL) for an additional 2 h period (19). Both untreated and occidiofungin-treated cells were fixed for 30 min in 4% formaldehyde. Cells were harvested by centrifugation (2,300× *g* for 5 min at 20°C), washed twice with 1× PBS, and fixed in 4% formaldehyde in 1× PBS for an additional 1 h with rocking at room temperature. Fixed cells were permeabilized in 0.1% TritonX-100 in 1× PBS for 1 h followed by two washes with 1× PBS. ActinGreen 488 stain was prepared by diluting two drops of solution in 500 µL of 1× PBS. Actin staining was carried out on permeabilized cells for 2 h at room temperature with gentle rocking. An aliquot of stained cells was removed and centrifuged for 5 min at 2,300× *g*. Cells were resuspended in a nominal volume of Vecta-Shield with DAPI (Vector Laboratories) and mounted on a poly-lysine-coated glass slide with a coverslip.

For biofilms, 24-h *Candida* biofilms were exposed to 0.5× MBIC_90_ occidiofungin for 3 h. Both untreated and occidiofungin-treated biofilms were stained with Live-or-Dye™ 640/662 Fixable Viability Stain (Biotium) for 30 min in PBS, washed twice, and fixed in 4% formaldehyde for 90 min. Fixed biofilms were washed twice with 1× PBS, permeabilized in 0.1% TritonX-100 in 1× PBS for 1 h followed by two washes with 1× PBS. ActinGreen 488 stain was prepared as described above. Actin staining was carried out on permeabilized biofilms in the presence of 50 µg/mL CW for 2 h. All steps for staining were performed at room temperature. Actin images were collected from a minimum of two independent biological experiments.

### Confocal laser scanning microscopy

Confocal microscopy was carried out using Leica SP8 CLSM with LAS X software. For imaging, stained biofilms were inverted onto a 35-mm diameter glass-bottom microwell dish (MatTek Corp) and switching cells were mounted on a polylysine-coated glass slides. Images were collected using 40× (1.15 NA) or 63× (1.3 NA) oil immersion objectives with 405 nm, 488 nm, and 635 nm lasers for CW or DAPI, Con A-FITC or ActinGreen 488, and Live-or-Dye™ 640/662 stains, respectively. Images were captured as z-stacks with a z-step size of 0.5 µm. A frame average of 4 was used and at least 10 random fields were captured and analyzed for each biofilm. Quantification of biofilms was done using the software BiofilmQ software using data collected from three independent experiments (48).

### Biofilm quantification using BiofilmQ

Quantitative analysis of biofilm structures was done using BiofilmQ (48). Each z-stack was first processed for segmentation using the automated thresholding method, Otsu. The accuracy of segmentation results was visually inspected in BiofilmQ and the thresholding manually adjusted using the sensitivity value. For quantification, a cube size of 9 voxels and 12 voxels was used for *C. albicans* and *C. tropicalis* as the average cell size for all biofilms. Global biofilm properties including the mean for biofilm thickness, biovolume, and ECM fluorescence intensity were then calculated.

Reduction in cell viability was quantified by comparing the biovolume measurements obtained from BiofilmQ analysis for two data images collected for each field: Calcofluor White (total cells) and Live-or-Dye™ 640/662 (dead cells) stained cells. The relative biovolume occupied by dead cells was determined by dividing the biovolume obtained for Live-or-Dye™ 640/662 by the biovolume obtained for Calcofluor White, with results reported as a percent. The extracellular matrix volume was calculated as the mean fluorescence intensity for Con A-FITC and reported in arbitrary units.

### Statistical analysis

The average and standard deviation obtained from independent experiments were analyzed using one-way ANOVA using post hoc Tukey HSD method to identify statistically significant differences between treated and untreated control samples. A *P*-value < 0.05 was considered statistically significant.

## Data Availability

All data supporting the findings of this study are available from the corresponding author upon request.

## SUPPLEMENTAL MATERIAL

Supplemental figures and table spectrum.02460-23-s0001.pdf Fig. S1, S2, S3 and Table S1, with accompanying Methods for supplemental data.

ASM does not own the copyrights to Supplemental Material that may be linked to, or accessed through, an article. The authors have granted ASM a non-exclusive, world-wide license to publish the Supplemental Material files. Please contact the corresponding author directly for reuse.
