# Supplementary material for: Occidiofungin inhibition of Candida biofilm formation on silicone elastomer surface
Source: Microbiol Spectr. 2023 Oct 10;11(6):e02460-23. doi: 10.1128/spectrum.02460-23 (PMC10715079; doi:10.1128/spectrum.02460-23)
Supplement: Supplemental figures and table — Fig. S1, S2, S3 and Table S1, with accompanying Methods for supplemental data. [file spectrum.02460-23-s0001.pdf]

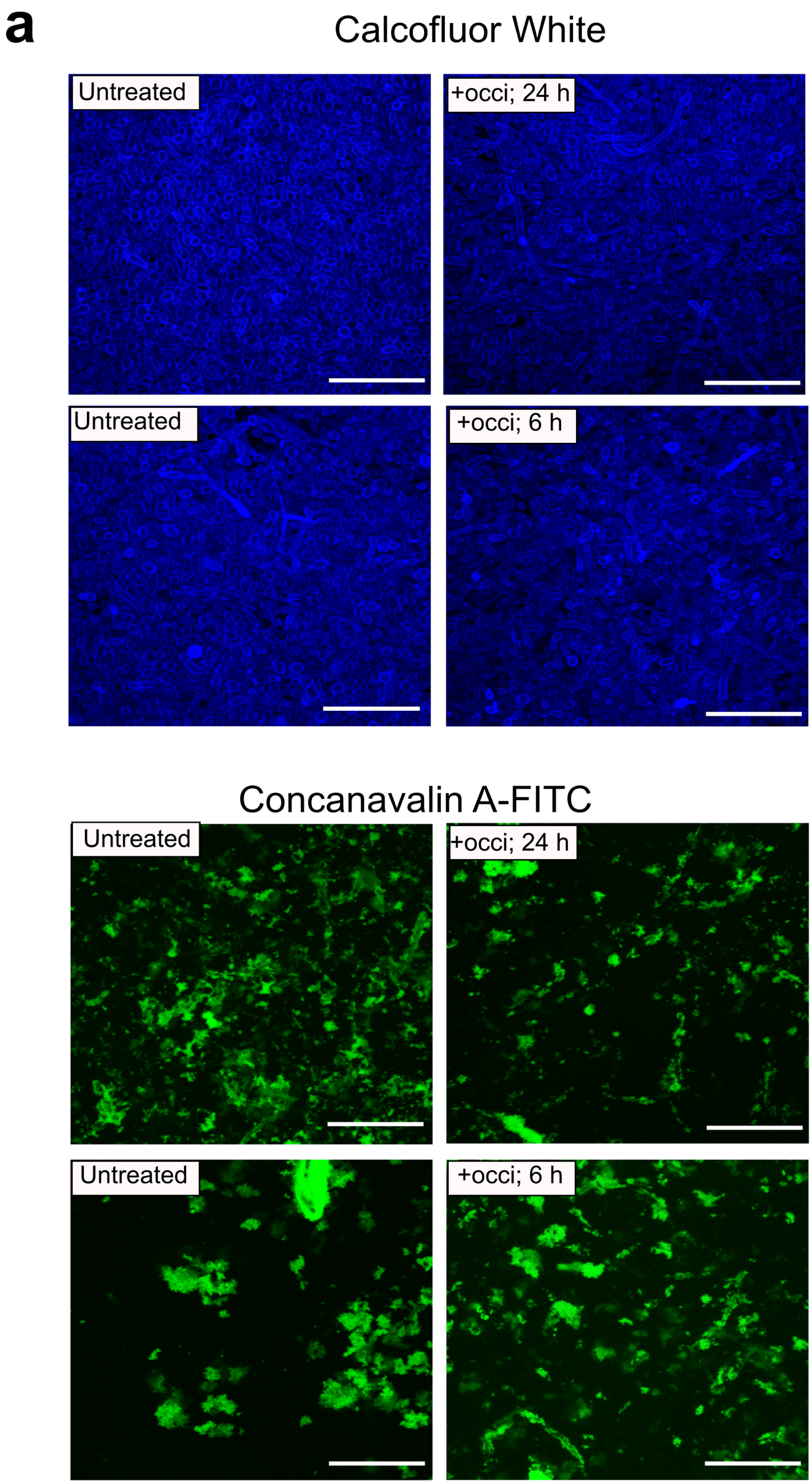
a
Calcofluor White
Untreated
+occi; 24 h
Untreated
+occi; 6 h
Concanavalin A-FITC
Untreated
+occi; 24 h
Untreated
+occi; 6 h

b

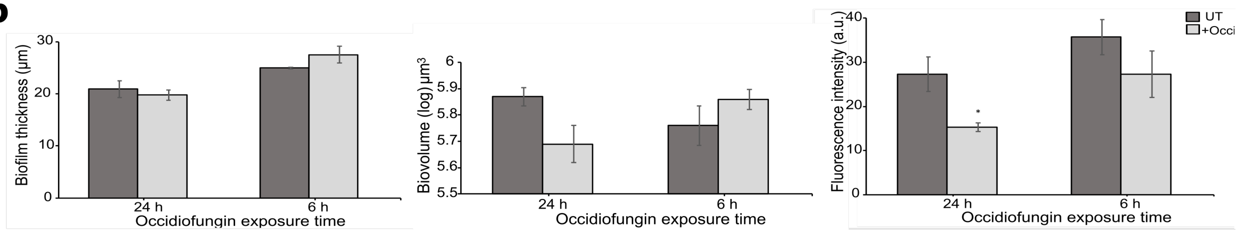

Fig. S1 Occidiofungin alters the morphology of biofilm cells. Organization of *C. tropicalis* ATCC 66024 cells in a preformed biofilm following growth in RPMI media (Untreated) or RPMI with 0.5X $MBIC_{90}$ occidiofungin (+Occ) for 6 h and 24 h. (a) Representative images of biofilms co-stained with Calcofluor White (left panel) and Concanavalin A-FITC (right panel) to observe cell morphology and extracellular matrix, respectively. Images are displayed as maximum intensity projections of 3D z-stacks. Size bar; 50 micron. (b) Biofilm thickness, biovolume, and mean fluoroscence intensity for ECM (Concanavalin A-FITC) are reported for untreated (dark bars) and occidiofungin treated (light bars) biofilms with data collected from 10 different fields for each biological replicate and 3 independent biological replicates per treatment condition. Data represents the average and standard error. Significant differences, as determined using post hoc Tukey HSD method, between untreated and occidiofungin exposed biofilms are indicated; * , $p < 0.05$.

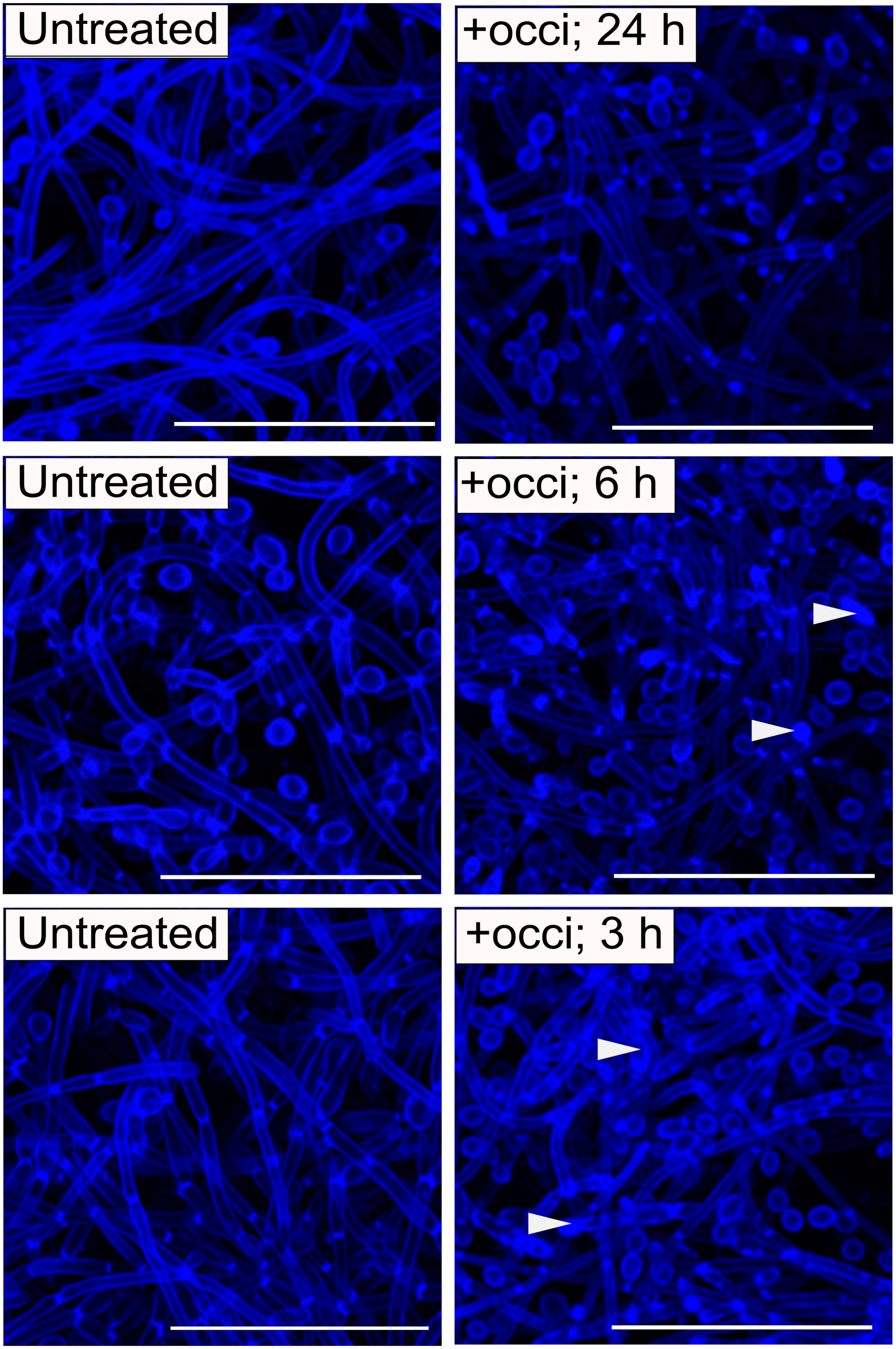
Untreated
+occi; 24 h
Untreated
+occi; 6 h
Untreated
+occi; 3 h

FIG S2 Occidiofungin alters chitin distribution in biofilm cells. A representative enlarged area from Figure 4 for *C. albicans* ATCC 66027 biofilm cells stained with Calcofluor White to observe cell morphology and chitin distribution. Images are displayed as maximum intensity projections of 3D z-stacks. White arrowhead; cell with increased chitin staining. Size bar; 50 micron.

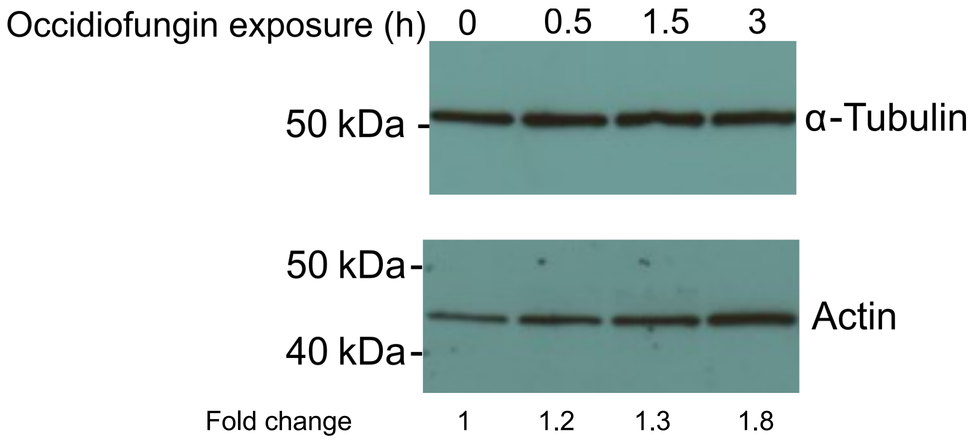

Fig. S3 No reduction in actin protein levels following occidiofungin exposure. *C. albicans* ATCC 66027 cells in a preformed biofilm in RPMI (0 h) or RPMI with 0.5X $MBIC_{90}$ occidiofungin for 0.5 h,1.5 h, and 3 h were analyzed for actin protein levels by immunoblot. Fold change in actin levels relative to alpha-tubulin (loading control) were normalized to that of T=0 h sample.

Table S1: Morphology of biofilm-dispersed cells

Strain	Morphology score (cell number/total)			
	Y/total	%	P-H/total	%
C. albicans **ATCC 66027**				
N=1	197/214	92.1	17/214	7.9
N=2	197/221	89.1	24/221	10.9
N=3	185/206	89.8	21/206	10.2
C. tropicalis **ATCC 66024**				
N=1	157/215	75.4	58/215	24.6
N=2	170/236	72.0	66/236	27.9
N=3	187/248	73.0	61/248	26.9

Abbreviations: Y, yeast; P-H, pseudohyphae or hyphae; N, biological replicate

Supplemental Material and Methods

Protein extraction and immunoblot

Preformed biofilms of *Candida albicans* ATCC 66027 developed on SE disks were exposed to DMSO (vehicle control) or 0.5X $MBIC_{90}$ (8 μg/ml) of occidiofungin for 0.5 h, 1.5 h, and 3 h at 37°C. Untreated and treated biofilms were disrupted and subjected to total protein extraction using the alkaline cell lysis method followed by TCA precipitation as described (49). Proteins separated by SDS-PAGE, were transferred to nitrocellulose membrane, probed with antibodies against β-actin (RRID: AB_626632) and α-tubulin (RRID: AB_1157911), and developed using HRP-conjugated secondary antibodies with Pierce Femto detection reagents. Autoradiographs were analyzed using NIH Image J software with differences in actin relative to tubulin normalized to that of the untreated sample.

Microscopy for dispersed cells

Cells released in media from a 48-h biofilm were fixed for 24 h in 3.7% formaldehyde. Fixed cells were visualized using a Nikon Eclipse-50 microscope under 100X (1.25 NA) oil immersion objective. Images captured using black and white Retiga Camera and QImaging software were scored as yeast, hyphae or pseudohyphae cells for at least 200 cells for each of three independent biological replicates.

Supplementary material: Confocal imaging settings

Conditions for imaging stained biofilm cells

Microscope name: Leica DMi SP8

Software: Leica Microsystems LAX software (version 3.5.7.23225)

Image size: 526 x 526 pixels

Step size: 0.5 microns

Detector: PMT

Frame average: 1

Dye names: Calcofluor White, Concanavalin A-FITC, ActinGreen 488, Live-or-Dye™ 640/662

Objective lens: APO ACS 40X/1.15 Oil

Scan speed: 400

Zoom: 1.5

Pinhole: 1

Image processing: Maximum intensity z-projection

Imaging temperature: 20 - 25°C

Conditions for imaging stained hyphal cells (single cells)

Microscope name: Leica DMi SP8

Software: Leica Microsystems LAX software (version 3.5.7.23225)

Image size: 1024 x 1024 pixels

Step size: 0.5 microns

Detector: PMT

Frame average: 4

Dye names: ActinGreen 488, DAPI

Objective lens: APO ACS 63X/1.3 Oil

Scan speed: 400

Zoom: 1.5

Pinhole: 1

Image processing: Maximum intensity z-projection

Imaging temperature: 20 - 25°C
